# GP Consultations for Venous Thromboembolism (VTE) After mRNA and Adeno‐Vector‐Based COVID‐19 Vaccination—An Exposure‐Anchored Self‐Controlled Cohort Study Based on Primary Healthcare Data From the Netherlands

**DOI:** 10.1002/pds.70317

**Published:** 2026-01-04

**Authors:** R. Jajou, E. P. van Puijenbroek, K. Hek, J. A. Overbeek, F. P. A. M. van Hunsel, Erik Mulder, A. C. Kant

**Affiliations:** ^1^ Netherlands Pharmacovigilance Centre Lareb 's‐Hertogenbosch the Netherlands; ^2^ Department of PharmacoTherapy, Epidemiology and Economics, Groningen Research Institute of Pharmacy (GRIP) University of Groningen Groningen the Netherlands; ^3^ Nivel (Netherlands Institute for Health Services Research) Utrecht the Netherlands; ^4^ PHARMO Institute for Drug Outcomes Research Utrecht the Netherlands; ^5^ Department of Clinical Pharmacy and Toxicology Leiden University Medical Center Leiden the Netherlands

**Keywords:** COVID‐19 vaccination, mRNA vaccination, primary healthcare data, self‐controlled cohort study, vector vaccination, venous thromboembolism

## Abstract

**Introduction:**

Venous thromboembolism (VTE) is labeled as an adverse effect of the adeno‐vector‐based vaccines AstraZeneca and Johnson & Johnson. We aimed to study whether there was an increase in general practitioner (GP) consultations for VTE after COVID‐19 vaccination.

**Methods:**

An exposure‐anchored self‐controlled cohort study was performed among COVID‐19 vaccinated persons aged ≥ 12 years who were registered in the PHARMO Data Network and Nivel Primary Care Database in the Netherlands. The focal window was set at 28 days after each COVID‐19 vaccination and the referent window at all time outside the focal window. Adjusted incidence rate ratios (aIRR), adjusting for SARS‐CoV‐2 infection, were calculated using Poisson regression.

**Results:**

In total, 2 133 853 persons were included. The highest increase in GP consultations for VTE was observed after Johnson & Johnson vaccination (aIRR: 3.14, 95% CI: 1.50–6.57), and a slight increase after Pfizer/BioNTech dose 1 (aIRR: 1.24, 95% CI: 1.09–1.40). Risk groups were 12–60 year‐olds with increased GP consultations for VTE after Johnson & Johnson (aIRR: 2.30, 95% CI: 1.44–3.69) and Pfizer/BioNTech (aIRR: 1.29, 95% CI: 1.11–1.50), and in specific groups of males aged 12–60 years. Also, females using hormone‐containing contraceptives or hormone replacement therapy (HRT) showed increased GP consultations for VTE after AstraZeneca (aIRR: 2.87, 95% CI: 1.13–7.33) and Pfizer/BioNTech (aIRR: 1.48, 95% CI: 1.10–2.01).

**Conclusion:**

Increased GP consultations for VTE were observed after both vector and mRNA vaccination, in particular among males, 12–60 year olds, and females using hormone‐containing contraceptives or HRT.

## Key Points

- Venous thromboembolism (VTE) is a relatively common condition where blood clots form in veins, and it has been noted as a possible adverse reaction of some COVID‐19 vaccines.
- This study investigated whether there was an increase in general practitioner (GP) consultations for VTE after COVID‐19 vaccination.
- An exposure‐anchored retrospective self‐controlled cohort study was performed among COVID‐19 vaccinated persons aged ≥ 12 years who were registered in two Dutch GP databases.
- The highest increase in GP consultations for VTE was observed after Johnson & Johnson vaccination (aIRR: 3.14, 95% CI: 1.50–6.57), and a slight increase after Pfizer/BioNTech dose 1 (aIRR: 1.24, 95% CI: 1.09–1.40).
- To better understand this risk, a more comprehensive review of all available studies is recommended, as current findings vary.

## 1 Introduction

Venous thromboembolism (VTE) refers to the occurrence of blood clots in the veins and includes deep vein thrombosis and pulmonary embolism [1, 2]. With deep vein thrombosis, the blood clot is formed in a deep vein, usually in the lower leg or in the arms. Pulmonary embolism occurs when a blood clot breaks off and travels through the blood to the lungs [1, 2]. Without treatment, VTE can restrict or block blood flow, which can damage organs or body tissue or can even lead to death due to limited oxygen supply [2]. Several risk factors contribute to developing VTE, such as surgery, major injury, infection, hormonal contraceptives and hormone replacement therapy (HRT), older age, immobility, or certain diseases like cancer, lung disease, and heart disease [1, 2]. VTE is a common disease with an incidence of 1–2 per 1000 person‐years in Western countries [3], and the incidence increases with age.

Shortly after the start of the COVID‐19 vaccination campaign, the first safety signals were raised regarding the occurrence of thrombosis after the adeno‐vector‐based (hereafter called “vector” vaccine) COVID‐19 vaccine from AstraZeneca [4, 5, 6]. This included a study from the Nordic countries, in which increased risks of venous thromboembolic events, including cerebral venous thrombosis, were observed after vaccination with the AstraZeneca vaccine [5]. VTE has been labeled as an adverse effect of the vector COVID‐19 vaccines of AstraZeneca and Johnson & Johnson [7, 8]. On March 27, 2024, the European Commission permanently withdrew the marketing authorization for AstraZeneca in the European Union at the request of the marketing authorization holder (MAH) due to commercial reasons (because of a decline in demand for AstraZeneca) [9]. Also, the marketing authorization for Johnson & Johnson has been withdrawn at the request of the MAH [10]. However, the risk of VTE after the mRNA vaccines Pfizer/BioNTech and Moderna is still unclear as published studies show non‐consistent results [4, 6, 11, 12, 13, 14, 15, 16, 17, 18, 19, 20, 21, 22, 23, 24, 25, 26, 27]. Several well designed and large sample sized studies showed an increased risk of VTE after the mRNA vaccines Pfizer/BioNTech and Moderna [12, 13, 14, 16, 18, 23], but other (among which also well designed and large sample sized) studies showed no increased risk [6, 11, 15, 17, 19, 20, 21, 22, 24, 26, 27, 28, 29, 30]. The studies regarding the mRNA vaccines were mainly published shortly after the rollout of the COVID‐19 vaccination, and only a few recent studies have been published. Also, in general, the majority of studies (on both the vector and mRNA vaccines) did not perform a detailed analysis on the risk of VTE after COVID‐19 vaccination for relevant subgroups, such as sex, age, and chronic illnesses.

In the current study, we aimed to use routine general practitioner (GP) healthcare data from the Netherlands to investigate whether there was an increase in GP consultations for VTE after COVID‐19 vaccination and to identify potential risk groups with increased GP consultations for VTE after COVID‐19 vaccination. This study was part of a pilot project in which an infrastructure was built to perform in‐depth analyses on several potential adverse drug reactions (ADRs) of COVID‐19 vaccination based on Electronic Health Record (EHR) data.

## 2 Methods

### 2.1 Study Population

We included persons aged ≥ 12 years who were registered in the Nivel Primary Care Database (Nivel‐PCD) or the PHARMO Data Network on January 1, 2016, and who had received ≥ 1 COVID‐19 vaccination in 2021. Persons who consulted the GP for VTE in the 5 years prior to cohort entry (thus between January 1, 2016, and December 31, 2020) were excluded so that only incident VTE cases were included.

### 2.2 GP Databases

PHARMO's GP data covers approximately 20% of the Dutch population [31] and the Nivel‐PCD 8%–10% [32]. Of the persons aged ≥ 12 years from 2021, approximately 5% was overlapping, that is, present in both GP databases. If a person's data were present in both the Nivel‐PCD and PHARMO databases, the information from the PHARMO database was used, as it also uses free text fields to collect data, which could result in more complete data. Information about age, sex, SARS‐CoV‐2 infection, preselected comorbidities, and potential risk groups were obtained from the PHARMO and Nivel‐PCD GP databases and were collected over the years 2016–2021. These variables (except age and sex) were defined based on International Classification of Primary Care (ICPC) codes used to code symptoms or diagnoses by GPs, Anatomical Therapeutic Chemical (ATC) codes used to code the drugs that are used, or a combination of ICPC and ATC codes (see Table S1).

### 2.3 Outcome Definition

VTE was defined as being diagnosed with pulmonary embolism or deep vein thrombosis. Due to pulmonary embolism often occurring after deep vein thrombosis, both diagnoses were analyzed together under the definition of VTE. The following ICPC codes were collected from the GP databases to define the outcome VTE: K94.01 deep vein thrombosis in the leg, K93 pulmonary embolism/pulmonary infarction, and W77.03 deep vein thrombosis during pregnancy. Only the first GP consultation for VTE in 2021, which could be either ICPC code, was included in this study.

### 2.4 National Vaccination Database

The National Institute for Public Health and the Environment (RIVM) collects COVID‐19 vaccination data on a national level in the COVID Vaccination Information and Monitoring System (CIMS) for all persons who gave consent to share their data (approximately 94%) [33]. The CIMS database contains vaccinations that were administered during the pandemic by diverse institutions, including Municipal Health Services (GGD'en), GPs, and nursing homes. From the CIMS database, information on vaccination dates and vaccination brand per vaccination doses was collected over the year 2021. In addition, COVID‐19 vaccination data were also obtained from the PHARMO and Nivel‐PCD GP databases to supplement the CIMS data. For the overlapping COVID‐19 vaccination data, that is, vaccination data available in both the CIMS and GP database for the same person, the vaccination data from CIMS was used. See Table S2 for the criteria applied to clean the COVID‐19 vaccination data.

### 2.5 Study Design

An exposure‐anchored self‐controlled cohort study was performed. This design shares conceptual foundations with Self‐controlled Crossover Observational PharmacoEpidemiologic (SCOPE) studies [34, 35]. All persons were followed from the cohort entry date until the cohort exit date. The cohort entry date was January 1, 2021 and was the same for all persons. The cohort exit date could be reached at several points and could be different for each person. The cohort exit date was reached on the deregistration date at the GP practice in 2021 (e.g., due to moving or death of the patient) or on December 31, 2021, whichever cohort exit date came first. The follow‐up time was then divided into the focal window or exposed period (28 days after each vaccine dose) and the referent window or nonexposed period (the follow‐up period minus the exposed period). The 28‐day focal window was chosen in line with previous studies [4, 5, 6, 12, 13, 17, 18, 21, 22, 23, 24, 25, 28, 30]. If the consecutive vaccination was given in less than 28 days, the focal window of the prior vaccination stopped at the time the consecutive vaccination was administered. All individuals in our cohort must have received at least one vaccination.

### 2.6 Statistical Analyses

All results were analyzed in three COVID‐19 vaccination categories: all COVID‐19 vaccines together, by vaccine type (mRNA [i.e., Pfizer/BioNTech and Moderna] vs. vector [i.e., AstraZeneca and Johnson & Johnson]), and at product level by vaccine brand (Pfizer/BioNTech, Moderna, AstraZeneca, and Johnson & Johnson). For the analyses by vaccine type and vaccine brand, persons with a heterogeneous sequential vaccination regimen were excluded. These persons received different vaccine types or vaccine brands over time, and in case of overlapping vaccinations (i.e., < 28 days between vaccinations), it is difficult to determine with which vaccine type or vaccine brand VTE could be associated. In addition, results were shown for subgroup analysis by vaccine dose (doses 1–3) and by the following potential risk groups to investigate whether they had increased GP consultations for VTE after COVID‐19 vaccination compared to the control period: age (12–60/60+ years), sex (male/female), malignancy (yes/no), heart failure (yes/no), chronic lung disease (yes/no), and females using hormone‐containing contraceptives or HRT to treat common menopausal symptoms (yes/no). The age category 12–60 and 60+ years is based on the association between VTE and age, in which the risk of VTE strongly increases in individuals aged 60 years and older. For the analysis of the latter risk group, only females were included.

For each of the above analyses, incidence rates (per 100 000 persons‐years) were calculated for the focal window and referent window, after which the incidence rate ratio (95% CI) could be calculated using Poisson regression. The self‐controlled study design automatically controls for time‐invariant measured and unmeasured confounders, and only time‐varying confounders are necessary to control for. Therefore, we report adjusted incidence rate ratios (aIRR), adjusted for SARS‐CoV‐2 infection during the follow‐up period in 2021. SARS‐CoV‐2 infection was defined based on ICPC codes (see Table S1). Statistical significance was defined as *p* values below 0.05. RStudio version 4.2.2 was used to perform all data cleaning and data analysis.

### 2.7 Privacy

All the data described below were anonymized before they were shared for data analysis. Obtaining informed consent from patients or approval by a medical ethics committee is not obligatory for observational studies containing no directly identifiable data in the Netherlands (Dutch Civil Law, Article 7: 458). The study was approved according to the governance code of Nivel‐PCD (number: NZR‐00322.008) and by the institutional review board of STIZON, Utrecht, the Netherlands (number: CC2022‐13).

## 3 Results

A total of *n* = 2 133 853 persons were included in the study, of which 88% (*n* = 1 876 958) were from PHARMO's GP data and 12% (*n* = 256 895) from the Nivel‐PCD database. The median (interquartile range [IQR]) age of the study population was 51 (IQR = 32) years, and the proportion of males and females was equal (49.4% males vs. 50.6% females). Cardiovascular disease and chronic lung disease were the most common comorbidities. The majority of persons (54.4%) received two COVID‐19 vaccinations, which were mostly Pfizer/BioNTech. Based on vaccine type (vector vs. mRNA vaccine), *n* = 1 951 894 (91.5%) persons received a homogeneous sequential vaccination (same vaccine platforms are used across doses to achieve immunization) regimen and based on vaccine brand, *n* = 1 526 314 (71.5%) persons received a homogeneous sequential vaccination regimen (see Table 1).

**TABLE 1:** Characteristics of *N* = 2 133 853 COVID‐19 vaccinated persons aged ≥ 12 years.

	Total study population (*N* = 2 133 853)
Source population, *n* (%)	
PHARMO GP data	1 876 958 (88%)
Nivel‐PCD	256 895 (12%)
Age, median (IQR)	51 (32)
Age categories (in years), *n* (%)	
12–18	81 396 (8.5%)
19–39	511 394 (24%)
40–60	736 851 (34.5%)
60+	704 212 (33%)
Sex, *n* (%)	
Male	1 053 702 (49.4%)
Female	1 080 151 (50.6%)
Comorbidity, *n* (%)	
Cardiovascular disease	544 305 (25.5%)
Chronic lung disease	348 847 (16.3%)
Diabetes type 1 and 2	156 019 (7.3%)
Malignancy	117 308 (5.5%)
Psoriasis	48 475 (2.3%)
Obesity	34 868 (1.6%)
Inflammatory bowel disease	14 927 (0.7%)
HIV	3382 (0.2%)
Chronic kidney disease	2670 (0.1%)
Number of COVID‐19 vaccinations per person, *n* (%)	
1	230 329 (10.8%)
2	1 159 897 (54.4%)
3	743 556 (34.8%)
4	71 (0%)
Follow‐up time during the study period in 2021, *n* (%)	
≤ 3 months	25 999 (1.2%)
Between 3 and ≤ 6 months	28 111 (1.3%)
Between 6 and ≤ 9 months	26 056 (1.2%)
Between 9 and ≤ 12 months	2 053 687 (96.2%)
Total COVID‐19 vaccinations per vaccine brand, *n* (%)	
Pfizer/BioNTech	3 308 512 (69.2%)
Moderna	887 470 (18.6%)
AstraZeneca	453 687 (9.5%)
Johnson & Johnson	120 605 (2.5%)
Unknown	10 801 (0.2%)
Total number of persons with a heterogeneous/homogeneous sequential vaccination regimen based on vaccine type (mRNA vs. vector), *n* (%)	
Heterogeneous	181 959 (8.5%)
Homogeneous	1 951 894 (91.5%)
Total number of persons with a heterogeneous/homogeneous sequential vaccination regimen based on vaccine brand (Pfizer/BioNTech, Moderna, AstraZeneca, and Johnson & Johnson), *n* (%)	
Heterogeneous	607 539 (28.5%)
Homogeneous	1 526 314 (71.5%)

### 3.1 GP Consultations for VTE After COVID‐19 Vaccination, by Vaccine Type, Vaccine Brand, and Vaccine Dose

**FIGURE 1:**
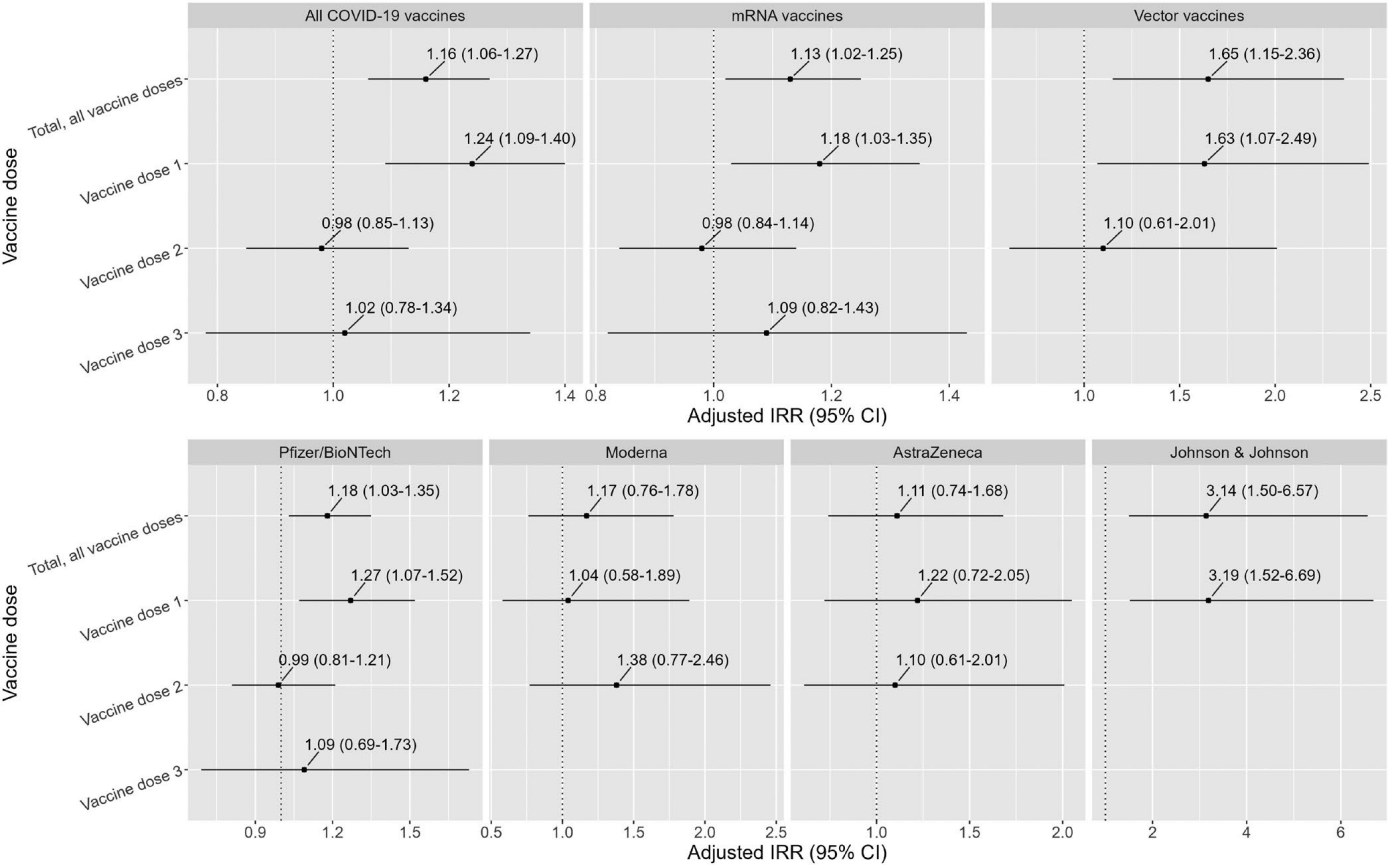
GP consultations for VTE after COVID‐19 vaccination by vaccine dose, vaccine type, and vaccine brand. The dotted line represents an adjusted IRR of 1 (= no effect, meaning no increase or decrease in GP consultations). Blank results represent cell counts below 5 and are not shown. For this reason, results related to the second dose of the Johnson & Johnson vaccine or the third dose of the Johnson & Johnson, Moderna, or AstraZeneca vaccine are not shown.

An increase in GP consultations for VTE was observed for all vaccine doses as well as for Dose 1 after COVID‐19 vaccination, mRNA vaccination, vector vaccination, Pfizer/BioNTech vaccination, and Johnson & Johnson vaccination. The highest increase in GP consultations for VTE was observed after Johnson & Johnson vaccination (aIRR: 3.14, 95% CI: 1.50–6.57) and in line with this, after vaccination with a vector vaccine (aIRR: 1.65, 95% CI: 1.15–2.36), in which both results represent all vaccine doses analyzed together. In addition, an increase in GP consultations for VTE was observed after Dose 1 of the Pfizer/BioNTech vaccine (aIRR: 1.27, 95% CI: 1.07–1.52), which represents the increase after COVID‐19 vaccination Dose 1 (aIRR: 1.24, 95% CI: 1.09–1.40) (see Figure 1 and Table S3). No increase in GP consultations for VTE was observed for any of the doses of Moderna and AstraZeneca.

### 3.2 GP Consultations for VTE After COVID‐19 Vaccination, by Sex and Age

**FIGURE 2:**
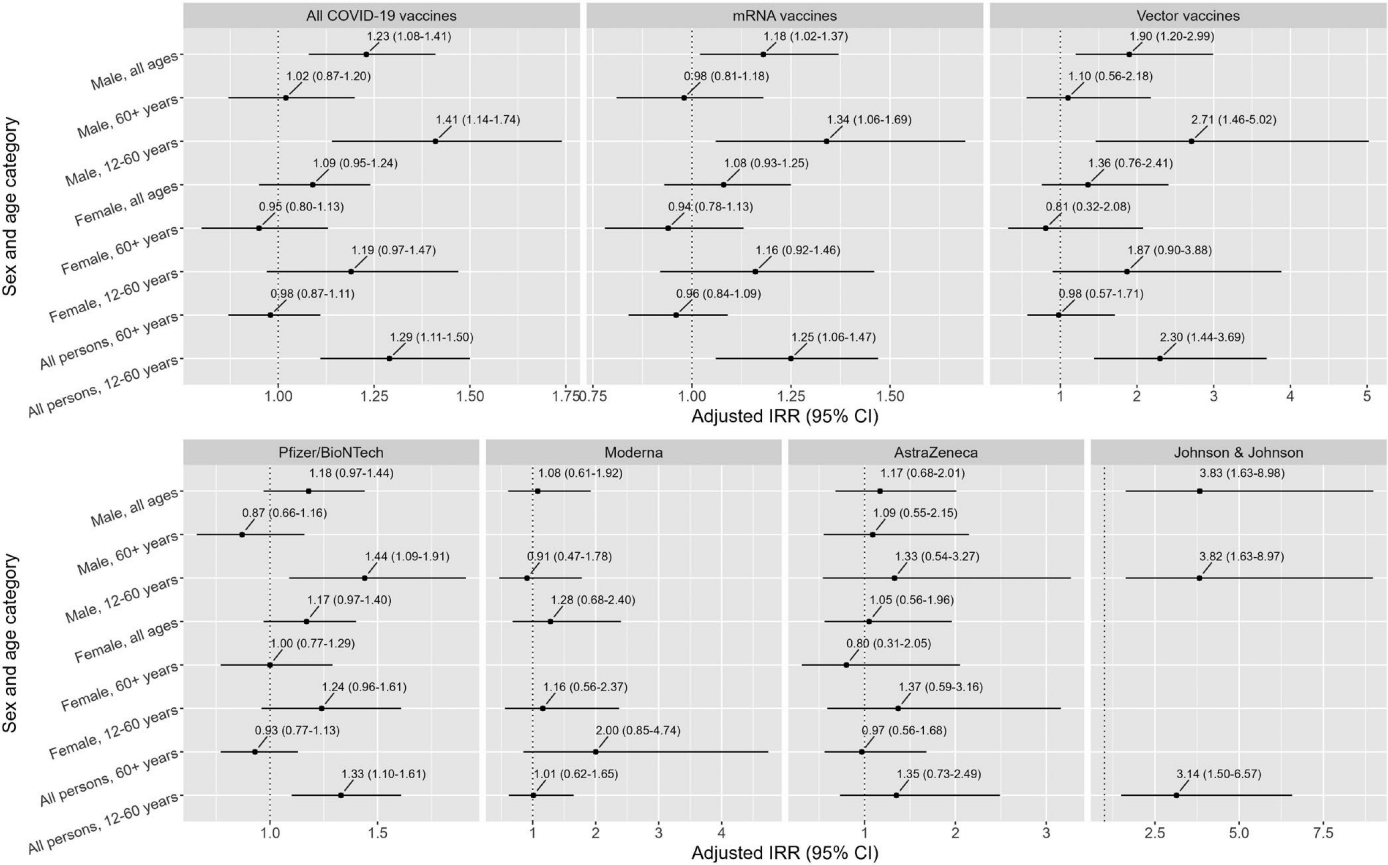
GP consultations for VTE after COVID‐19 vaccination by sex, age category, vaccine type, and vaccine brand. The dotted line represents an adjusted aIRR of 1 (= no effect, meaning no increase or decrease in GP consultations). Blank results represent cell counts below 5 and are not shown.

Stratifying the results by age category showed an increase in GP consultations for VTE for persons aged 12–60 years after both vaccination with a vector vaccine and an mRNA vaccine, with a higher increase after vector vaccination. The highest increase in GP consultations for VTE was seen among 12–60 year olds after Johnson & Johnson vaccination (aIRR: 3.14, 95% CI: 1.50–6.75). This vaccine was also administered to the younger age group (see Table S4). An increase in GP consultations for VTE among 12–60 year olds was also observed after Pfizer/BioNTech vaccination (aIRR: 1.33, 95% CI: 1.10–1.6160). No increase in GP consultations for VTE was observed among persons older than 60 years (for none of the vaccine types or vaccine brands), nor after Moderna and AstraZeneca vaccination (for none of the age groups) (see Figure 2 and Table S4).

Stratifying the results by sex and age category showed an increase in GP consultations for VTE for males aged 12–60 years after both vector and mRNA vaccination, in which also here the highest increase was observed after Johnson & Johnson (aIRR: 3.82, 95% CI: 1.63–8.97), followed by Pfizer/BioNTech (aIRR: 1.44, 95% CI: 1.09–1.91). In addition, males of all ages showed an increase in GP consultations for VTE after Johnson & Johnson (aIRR: 3.83, 95% CI: 1.63–8.98), and after both vector (aIRR: 1.90, 95% CI: 1.20–2.99) and mRNA vaccination (aIRR: 1.18, 95% CI: 1.02–1.37). No increase in GP consultations for VTE was observed among females (for none of the age groups, vaccine types, and vaccine brands) (see Figure 2 and Table S4).

### 3.3 GP Consultations for VTE After COVID‐19 Vaccination, by Risk Groups

**FIGURE 3:**
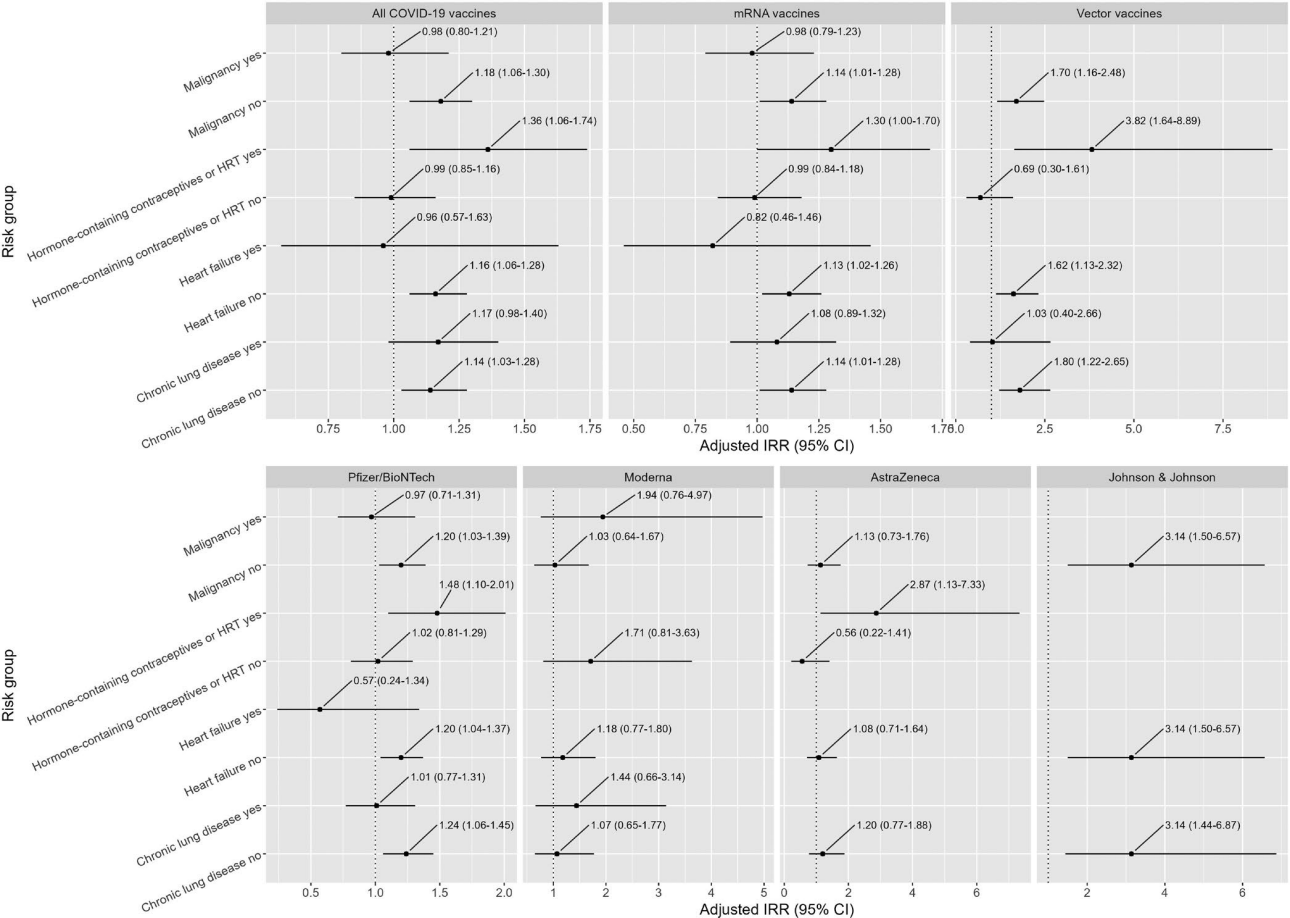
GP consultations for VTE after COVID‐19 vaccination by risk group, vaccine type, and vaccine brand. The dotted line represents an adjusted aIRR of 1 (= no effect, meaning no increase or decrease in GP consultations). Blank results represent cell counts below 5 and are not shown.

An increase in GP consultations for VTE was observed after COVID‐19 vaccination in general for persons without a malignancy (aIRR: 1.18, 95% CI: 1.06–1.30), heart failure (aIRR: 1.16, 95% CI: 1.06–1.28), or a chronic lung disease (aIRR: 1.14, 95% CI: 1.03–1.28) (see Figure 3 and Table S5).

With respect to vector and mRNA vaccine types, an increase in GP consultations for VTE was seen for persons vaccinated with a vector vaccine without malignancy (aIRR: 1.70, 95% CI: 1.16–2.48), heart failure (aIRR: 1.62, 95% CI: 1.13–2.32), or a chronic lung disease (aIRR: 1.80, 95% CI: 1.22–2.65). A similar pattern was observed for the mRNA vaccines, where persons without malignancy (aIRR: 1.14, 95% CI: 1.01–1.28), heart failure (aIRR: 1.13, 95% CI: 1.02–1.26), or a chronic lung disease (aIRR: 1.14, 95% CI: 1.01–1.28) showed increased risks (see Figure 3 and Table S5).

The Moderna vaccine did not show increased risks for any of the risk groups, but vaccination with the Pfizer/BioNTech vaccination was associated with an increased risk in persons without malignancy (aIRR: 1.20, 95% CI: 1.03–1.39), heart failure (aIRR: 1.20, 95% CI: 1.04–1.37), or a chronic lung disease (aIRR: 1.24, 95% CI: 1.06–1.45). For the Johnson & Johnson vaccination, in persons without malignancy or heart failure, the risk of VTE was increased (aIRR: 3.14; 95% CI: 1.50–6.57), as well as for persons without a chronic lung disease (aIRR: 3.14; 95% CI: 1.44–6.87) (see Figure 3 and Table S5).

Females using hormone‐containing contraceptives or HRT had an increase in GP consultations for VTE after Pfizer/BioNTech (aIRR: 1.48, 95% CI: 1.10–2.01) and the AstraZeneca vaccine (aIRR: 2.87, 95% CI: 1.13–7.33), which is reflected in an increase in the risk after mRNA vaccines and vector vaccines.

## 4 Discussion

In this large cohort study based on primary healthcare data from the Netherlands, an increase in GP consultations for VTE was observed after vaccination with both the vector and mRNA vaccines, in which the increase after vector vaccination was higher. Risk groups with an increase in GP consultations for VTE after COVID‐19 vaccination were males, persons aged 12–60, females using hormone‐containing contraceptives or HRT, and individuals without malignancy, heart failure, or a chronic lung disease.

VTE is labeled as an adverse effect of the vector vaccines AstraZeneca and Johnson & Johnson [7, 8], but not of the mRNA vaccines Pfizer/BioNTech and Moderna [36, 37]. In line with our research, several other studies have been published that also showed an increased risk of VTE after administration of mRNA vaccines [12, 13, 14, 16, 18, 23]. Studies showed that the risk of VTE after mRNA vaccination was in general lower compared to the risk of VTE after vector vaccination, which was also observed in our study. For example, a recently published cohort study investigated the risk of different types of thrombosis after COVID‐19 vaccination among 99 million vaccinated people from eight countries worldwide. This study was based on healthcare data and showed an increased risk of pulmonary embolism after both vaccination with the AstraZeneca vaccine (OE ratio: 1.88, 95% CI: 1.32–2.58 after the third dose) and after vaccination with the mRNA vaccines Pfizer/BioNTech (OE ratio: 1.29, 95% CI: 1.26–1.32 after the first dose) and the Moderna vaccine (OE ratio: 1.33, 95% CI: 1.26–1.40 after the first dose) [14]. Another example is a self‐controlled case series study from the Nordic countries (Norway, Finland, and Denmark) which showed an increased risk of VTE after AstraZeneca vaccination (rate ratio: 1.83, 95% CI: 1.56–2.15) [23]. The same study also showed an increased risk of VTE after mRNA vaccination, but these risks were lower (rate ratio: 1.13, 95% CI: 1.07–1.20 for Pfizer/BioNTech and rate ratio: 1.21, 95% CI: 1.02–1.44 after Moderna).

A relatively larger number of studies, however, did not find an increased risk of VTE after mRNA vaccination [6, 11, 15, 17, 19, 20, 21, 22, 24, 26, 27, 28, 29, 30]. For example, two cohort studies from the same consortium among approximately 46 million adults from England used electronic health record data and showed no increased risk for VTE after mRNA vaccination, and also not after AstraZeneca vaccination [29, 30]. Instead, these studies showed significant protective effects of VTE after Pfizer/BioNTech, Moderna, and AstraZeneca. Although the crude analyses showed increased risks of VTE after vaccination with Pfizer/BioNTech or AstraZeneca compared to unvaccinated persons/pre‐vaccination period, these risks were attenuated after adjusting for age and sex and further attenuated after adjusting for other confounders [30]. A sub‐analysis showed an increased risk of VTE among persons younger than 50 years only after AstraZeneca (in the 1–28 days post‐vaccination) and not after Pfizer/BioNTech vaccination [30]. Due to these conflicting results between studies (also between studies that used similar source data, that is, healthcare data, and large sample sizes), it is difficult to draw clear conclusions on the risk of VTE after mRNA vaccination. Therefore, it is recommended that future research focus on combining all results from different studies by performing a systematic review and meta‐analysis.

To provide more detailed information regarding the risk of VTE after COVID‐19 vaccination, we have performed several sub‐analyses. Due to this, there is a possibility that some of the significantly increased results were found by chance. Although there are various methods to correct for this, we did not correct for multiple testing, which is in line with the majority of other studies that showed an increased risk of VTE after mRNA vaccination [12, 13, 14, 18]. However, even if we had not performed a large number of tests and had solely performed the main analyses (Figure 1), our conclusion of an increased risk of VTE after both vector vaccination and mRNA vaccination would still be valid.

For both vector and mRNA vaccines, increased risks of GP consultations for VTE were observed among individuals *without* malignancy [38], heart failure [39], or chronic lung disease [40]. Risks in this group were more pronounced for vector vaccines than for mRNA vaccines. Within the mRNA group, the Moderna vaccine showed no increased risks, whereas Pfizer/BioNTech was associated with modest elevations. The Johnson & Johnson vaccine demonstrated the strongest associations, with more than threefold increased risks in the absence of malignancy, heart failure, or chronic lung disease.

While there are more relevant comorbidities with a potential increased risk of VTE after COVID‐19 vaccination, for example, inflammatory bowel disease, we limited our risk group analyses to a few relevant comorbidities (malignancy, chronic lung disease, and heart failure). This is because conditions that are mainly treated in secondary care are likely to be underreported in GP databases, as was shown for malignancy [41]. Also, GPs might not always (correctly) register such comorbidities in the GP system when reported back from secondary care. The number of people with a malignancy, chronic lung disease, or heart disease was also relatively low in our study; therefore, the results for the group with those diseases could not always be shown (see blank results in Figure 3), which makes it difficult to draw clear conclusions from these analyses.

Already in March 2021, some European countries suspended the use of the AstraZeneca vaccine [42], and in April 2021, the EMA concluded that the risk for thrombosis should be listed as an adverse effect of the Johnson & Johnson vaccine [43]. Individuals with underlying conditions that increase the risk of VTE might have refrained from vaccination because of this.

The risk group analysis also showed increased risks of VTE after Pfizer/BioNTech, AstraZeneca, and vector vaccination among females using hormone‐containing contraceptives or HRT. Females using hormone‐containing contraceptives or HRT were defined based on having at least one respective ICPC or ATC code (see Table S2) registered in the GP database between 2016 and 2020. It is possible that with this definition, also past users were included. Therefore, we performed a sensitivity analysis with an adjusted definition, in which for all ICPC codes (except morning‐after spiral and contraception intrauterine) and ATC codes (except progestogen‐containing IUD), only the GP registration date in the 6 months before cohort entry was included. This sensitivity analysis showed similar results (data not shown), except that the risk was much higher among females using hormone‐containing contraceptives or HRT who received a vector vaccine (aIRR: 6.84, 95% CI: 2.11–22.19).

Our study has several strengths. First, we combined two large GP databases from the Netherlands, which allowed for more statistical power, a more representative geographic distribution of included persons, and a better representativeness of the Dutch population [31, 32, 44]. Second, using a self‐controlled cohort design allows for an automatic correction for all fixed and unknown/unmeasured confounders. This means that it also automatically corrects for differences between populations (such as age and comorbidities), which defined when and for which vaccine brand a population or person was eligible for during the COVID‐19 vaccination campaign. However, it remained necessary to correct for potentially relevant time‐varying confounders, such as SARS‐CoV‐2 infection, which we corrected for in our study. It should be noted that there have been studies that found that VTE incidence exhibits seasonal variation [45, 46, 47]. In our study, we have not corrected the results for seasonal effects.

A limitation of our study design is the lack of a defined risk interval for SARS‐CoV‐2 infection and the fact that SARS‐CoV‐2 infection is likely underreported in GP databases due to a person needing to give permission to share this data with their GP, or a positive test taken at home may not always have been confirmed with a PCR test, or not everyone might have been tested (due to being asymptomatic or even if persons had symptoms, they may have chosen not to get tested). Another limitation is that there could be selection bias among individuals who consented to share their vaccination data in the CIMS COVID‐19 database and/or selection bias in individuals who got vaccinated (compared to individuals who did not get vaccinated). However, selection bias is likely limited as CIMS misses only around 6% of the vaccinated persons who did not give permission to share their data [48]. Furthermore, since this study is based on ICPC codes in primary healthcare data, it relies on the accuracy of coding by GPs and the consistency of coding between GPs, which could be subject to information bias. We have included GP consultations with an underlying ICPC code for VTE. Dutch GPs should only use this code in case of a confirmed diagnosis. The NHG (Dutch College of General Practitioners) guidelines are evidence‐based clinical guidelines developed for GPs in the Netherlands. They provide standardized recommendations for diagnosing, treating, and managing various medical conditions in primary care. The NHG Guideline Deep Vein Thrombosis and Pulmonary Embolism [49] provides further diagnostics after anamnesis and physical examination, at the very least including additional laboratory testing (D‐dimer). It can never be completely ruled out that there may be an incorrect registration of the ICPC code, but the diagnostics are reasonably robust. In addition, VTE cases might have been missed in GP databases in case a VTE diagnosis was made in a hospital, and this was not reported back to the GP, or the GP did not (accurately) code this in the GP system. A medical chart review was not part of our study. Our study design assumes that if consecutive vaccinations occur within 28 days, the exposed period for the prior dose ends when the next dose is given. In theory, this approach may lead to overlapping risk intervals, potentially inflating the observed risk for the next dose, although we did not find a significantly increased risk after the second or third dose. Additionally, individuals who experience an adverse event after the first dose may delay or skip subsequent doses, introducing bias [50].

In conclusion, our self‐controlled cohort study among COVID‐19 vaccinated persons aged 12 years and older showed an increase in GP consultations in the Netherlands for VTE after vaccination with a vector vaccine (specifically Johnson & Johnson) and an mRNA vaccine (specifically Pfizer/BioNTech). Mainly, the male sex, the younger age group (12–60 years), and females using hormone‐containing contraceptives or HRT showed an increase in GP consultations for VTE after COVID‐19 vaccination. It is important to closely follow future studies that focus on the risk of VTE after mRNA vaccination, as several studies showed evidence of a possible relation, and mRNA vaccines are still used in current COVID‐19 vaccination campaigns.

### 4.1 Plain Language Summary

Venous thromboembolism (VTE) is a relatively common condition where blood clots form in veins, and it has been noted as a possible side effect of some COVID‐19 vaccines, like AstraZeneca and Johnson & Johnson. This study investigated whether there was an increase in people visiting their general practitioner (GP) for VTE after getting vaccinated with a COVID‐19 vaccine in the Netherlands. Medical records from over 2.1 million people in the Netherlands who were vaccinated against COVID‐19 were used. The number of GP visits for VTE in the 28 days after vaccination (focal window) to visits outside this timeframe (referent window) was compared. In the study design, the researchers also accounted for COVID‐19 infections, which can also increase the risk of VTE. In this large self‐controlled cohort study based on primary healthcare data from the Netherlands, an increase in GP consultations for VTE was observed after vaccination with both the adeno‐vector‐based vaccines (such as AstraZeneca and Johnson & Johnson) and mRNA vaccines (such as Pfizer), in which the increase after vector vaccination was higher. Risk groups with an increase in GP consultations for VTE after COVID‐19 vaccination were males, persons aged 12–60, and females using hormone‐containing contraceptives or hormone replacement therapy (HRT). To better understand this risk, a more comprehensive review of all available studies is recommended, as current findings vary.

## Funding

This research was part of a project that received funding from the Ministry of Health, Welfare and Sport (grant number SP332956). The funders had no role in the study design, data collection and analysis, decision to publish, or preparation of the manuscript.

## Conflicts of Interest

The authors R.J., E.P.v.P., F.P.A.M.v.H., E.M., and A.C.K. have no conflicts of interest. K.H. received funding from Pfizer for research (starting on December 1, 2024) not related to this study. J.A.O. is an employee of the PHARMO Institute for Drug Outcomes Research. This independent research institute performs financially supported studies for the government and related health care authorities and several pharmaceutical companies.

## Supporting information


**Table S1:** Definition of covariates and risk groups based on ICPC codes or a combination of ICPC and ATC codes, extracted from the GP systems over the years 2016–2021.
**Table S2:** Criteria applied to clean the COVID‐19 vaccination data received from the national COVID Vaccination Information and Monitoring System (CIMS) and the general practitioner (GP) databases.
**Table S3:** Incidence rates (95% CI) of VTE in the exposed and nonexposed period by doses, vaccine type, and vaccine brand.
**Table S4:** Incidence rates (95% CI) of VTE in the exposed and nonexposed period by sex, age category, vaccine type, and vaccine brand.
**Table S5:** Incidence rates (95% CI) of VTE in the exposed and nonexposed period by risk group, vaccine type and vaccine brand.
