# Supplementary material for: GP Consultations for Venous Thromboembolism (VTE) After mRNA and Adeno‐Vector‐Based COVID‐19 Vaccination—An Exposure‐Anchored Self‐Controlled Cohort Study Based on Primary Healthcare Data From the Netherlands
Source: Pharmacoepidemiol Drug Saf. 2026 Jan 4;35(1):e70317. doi: 10.1002/pds.70317 (PMC12765587; doi:10.1002/pds.70317)
Supplement: Supplementary file 1 — Table S1: Definition of covariates and risk groups based on ICPC codes or a combination of ICPC and ATC codes, extracted from the GP systems over the years 2016–2021. Table S2: Criteria applied to clean the COVID‐19 vaccination data received from the national COVID Vaccination Information and Monitoring System (CIMS) and the general practitioner (GP) databases. Table S3: Incidence rates (95% CI) of VTE in the exposed and nonexposed period by doses, vaccine type, and vaccine brand. Table S4: Incidence rates (95% CI) of VTE in the exposed and nonexposed period by sex, age category, vaccine type, and vaccine brand. Table S5: Incidence rates (95% CI) of VTE in the exposed and nonexposed period by risk group, vaccine type and vaccine brand. [file PDS-35-e70317-s001.docx]

**SUPPLEMENTARY MATERIAL**

**Supplementary table 1** Definition of covariates and risk groups based on ICPC codes or a combination of ICPC and ATC codes, extracted from the GP systems over the years 2016-2021.

| **Covariate / risk group** | **ICPC code** | **ATC code** |
| --- | --- | --- |
| SARS-CoV-2 infection | R83.03 SARS-CoV-2 (COVID-19)  R83.04 Long COVID-19 | NA |
| Hormone containing contraceptives or hormone replacement therapy | W10 Morning after pill / Contraception postcoital  W10.01 Morning after pill  W10.02 Morning after spiral  W11 Contraception oral  W12 Contraception intrauterine  W14 Contraception other female | G03C Estrogens  G03CA Natural and semisynthetic estrogens, plain  G03CB Synthetic estrogens, plain  G03CC Estrogens, combinations with other drugs  G03CX Other estrogens  G03AA Progestogens and estrogens, fixed combinations  G03AB Progestogens and estrogens, sequential preparations  G03F Progestogens and estrogens in combination  G03FA Progestogens and estrogens, fixed combinations  G03FB Progestogens and estrogens, sequential preparations  G03HB Antiandrogens and estrogens  G03HB01 Cyproterone and estrogen  L02AA Estrogens  G02BA Intrauterine contraceptives  G02BA03 Plastic IUD with progestogen  G02BB Intravaginal contraceptives  G02BB01 Vaginal ring with progestogen and estrogen  G02BB02 Vaginal ring with progestogen |
| Malignancy | A79 Malignancy NOS  B72 Hodgkin's disease/lymphoma  B72.01 Hodgkin's disease  B72.02 Non-Hodgkin lymphoma  B73 Leukemia  B74 Malignant neoplasm blood other  B74.01 Multiple myeloma  D74 Malignant neoplasm stomach  D75 Malignant neoplasm colon/rectum  D76 Malignant neoplasm pancreas  D77 Malig. neoplasm digest other/NOS  D77.01 Malignancy esophagus  D77.02 Malignancy salivary glands  D77.03 Malignancy lip/mouth/tongue  D77.04 Malignancy liver/gallbladder/bile duct  F74.01 Neoplasm of eye/adnexa  H75.01 Neoplasm of ear  K72.01 Neoplasm cardiovascular  L71.01 Malignant neoplasm musculoskeletal  N74 Malignant neoplasm nervous system  R84 Malignant neoplasm bronchus/lung  R85 Malignant neoplasm respiratory, other  S77 Malignant neoplasm of skin  S77.02 Spinocellular/squamous cell carcinoma  S77.03 Malignant melanoma  S77.04 Kaposi's sarcoma  T71 Malignant neoplasm thyroid  U75 Malignant neoplasm of kidney  U76 Malignant neoplasm of bladder  U77 Malignant neoplasm urinary other  W72 Malignant neoplasm relate to pregnancy  X75 Malignant neoplasm cervix  X76 Malignant neoplasm breast female  X76.01 Adenocarcinoma breast woman  X77 Malignant neoplasm genital female other  X77.01 Endometrial carcinoma  X77.02 Malignancy ovary  Y77 Malignant neoplasm prostate  Y78 Malignant neoplasm male genital other  Y78.01 Malignancy penis  Y78.02 Malignancy testis  Y78.03 Malignancy breast | L01A Alkylating agents  L01AA Nitrogen mustard analogues  L01AB Alkyl sulfonates  L01AC Ethylene imines  L01AD Nitrosoureas  L01AG Epoxides  L01AX Other alkylating agents  L01B Antimetabolites  L01BA Folic acid analogues  L01BB Purine analogues  L01BC Pyrimidine analogues  L01C Plant alkaloids and other natural products  L01CA Vinca alkaloids and analogues  L01CB Podophyllotoxin derivatives  L01CC Colchicine derivatives  L01CD Taxanes  L01CE Topoisomerase 1 (TOP1) inhibitors  L01CX Other plant alkaloids and natural products  L01D Cytotoxic antibiotics and related substances  L01DA Actinomycines  L01DB Anthracyclines and related substances  L01DC Other cytotoxic antibiotics  L01X Other antineoplastic agents  L01XA Platinum compounds  L01XB Methylhydrazines  L01XD Sensitizers used in photodynamic/radiation therapy  L01XF Retinoids for cancer treatment  L01XG Proteasome inhibitors  L01XH Histone deacetylase (HDAC) inhibitors  L01XJ Hedgehog pathway inhibitors  L01XK Poly (ADP-ribose) polymerase (PARP) inhibitors  L01XX Other antineoplastic agents  L01XY Combinations of antineoplastic agents  L02B Hormone antagonists and related agents  L02BA Anti-estrogens  L02BB Anti-androgens  L02BG Aromatase inhibitors  L02BX Other hormone antagonists and related agents |
| Heart failure | K77 Heart failure  K77.01 Acute congestive heart failure/cardiac asthma  K77.02 Chronic congestive heart failure  K77.03 Heart failure with preserved left ventricular ejection fraction  K77.04 Heart failure with moderate or reduced left ventricular ejection fraction | NA *(this is often a combination of different non-specific medications that can also be used for other conditions)* |
| Chronic lung disease | R91 Chronic bronchitis/bronchiectasis  R91.01 Chronic bronchitis  R91.02 Bronchiectasis  R95 Chronic obstructive pulmonary dis  R96 Asthma  R96.02 Allergic asthma  T99.10 Cystic fibrosis | R03 Drugs for obstructive airway diseases  R03A Adrenergics, inhalants  R03B Other drugs for obstructive airway diseases, inhalants  R03C Adrenergics for systemic use  R03D Other systemic drugs for obstructive airway diseases  R07AA Lung surfactants  R07AB Respiratory stimulants |
| ICPC: International Classification of Primary Care, ATC: Anatomical Therapeutic Chemical, NA: Not applicable | | |

**Supplementary table 2** Criteria applied to clean the COVID-19 vaccination data received from the national COVID Vaccination Information and Monitoring System (CIMS) and the General Practitioner (GP) databases.

| *Criteria 1* | All vaccinations are valid seven days before the campaign starts/a vaccine is put into use to build in a margin. Especially for the booster campaign, it is known that some institutions started vaccinating earlier than the start date of the campaign. |
| --- | --- |
| *Criteria 2* | There must be at least 21 days between vaccinations of the basic series (-7 days margin, i.e. 14 days between vaccinations is accepted) |
| *Criteria 3* | Starting date of the first two vaccinations of the basic series, by vaccine brand:   - Pfizer/unknown: January 4, 2021 (-7 days margin, i.e. January 1, 2021 is accepted) - Moderna: January 18, 2021 (-7 days margin, i.e. January 11, 2021 is accepted) - Astrazeneca: February 8, 2021 (-7 days margin, i.e. February 1, 2021 is accepted) - Johnson & Johnson: April 19, 2021 (-7 days margin, i.e. April 12, 2021 is accepted) - Pfizer bivalent: September 19, 2022 (vaccinations in 2021 are accepted) |
| *Criteria 4* | Starting date of the third vaccination of the basic series: October 1, 2021 (-7 days margin, i.e. September 24, 2021 is accepted). Only the Pfizer or Moderna vaccine (monovalent) is accepted for the third vaccination of the basic series. |
| *Criteria 5* | There must be at least 60 days between vaccinations from the basic series and the booster vaccination or between two booster vaccinations (-7 days margin, i.e. at least 53 days is accepted) |
| *Criteria 6* | Starting date of the booster vaccinations: November 18, 2021 (-7 days margin, i.e. November 11, 2021 is accepted). Only the Pfizer, Moderna or unknown brand is accepted for the booster vaccinations and only for persons aged 18 years or older. |

**Supplementary table 3** Incidence rates (95% CI) of VTE in the exposed and non-exposed period by doses, vaccine type and vaccine brand.

|  | **n** | **Person-years exposed** | **Events in exposed period**  **(IR per 100,000 person-years)** | **Person-years non-exposed** | **Events in non-exposed period (IR per 100,000 person-years)** |
| --- | --- | --- | --- | --- | --- |
| ***All COVID-19 vaccines (including unknown), n = 2,133,853 (total cohort)*** | | | | | |
| Total, all doses  Dose 1 ^1^  Dose 2 ^2^  Dose 3 ^3^ | 2,133,853  2,133,853  1,903,524  743,627 | 322,556  157,764  139,592  25,199 | 546 (169)  284 (180)  206 (148)  56 (222) | 1,753,785  1,753,785  1,5478,744  588,614 | 2695 (154)  2695 (154)  2457 (159)  1303 (221) |
| ***Vaccine type (mRNA versus vector), n = 1,951,894 persons with a homogeneous sequential vaccination regimen*** | | | | | |
| mRNA vaccines  Total, all doses  Dose 1 ^1^  Dose 2 ^2^  Dose 3 ^3^ | 1,769,141  1,769,141  1,640,645  607,363 | 272,980  130,376  121,181  21,422 | 453 (166)  225 (173)  176 (145)  52 (243) | 1,448,099  1,448,099  1,333,122  479,674 | 2244 (155)  2244 (155)  2077 (156)  1094 (228) |
| Vector vaccines  Total, all doses  Dose 1 ^1^  Dose 2 ^2^  Dose 3 ^3^ | 182,755  182,755  86,041  0 | 20,165  13,705  6,460  NA | 37 (183)  25 (182)  12 (186)  NA | 157,545  157,545  71,031  NA | 190 (121)  190 (121)  131 (184)  NA |
| ***Vaccine brand (Pfizer/BioNTech, Moderna, AstraZeneca, Johnson & Johnson), n = 1,526,314 persons with a homogeneous sequential vaccination regimen*** | | | | | |
| Pfizer/BioNTech  Total, all doses  Dose 1 ^1^  Dose 2 ^2^  Dose 3 ^3^ | 1,183,215  1,183,215  1,070,442  174,400 | 172,276  86,309  78,934  7,033 | 258 (150)  139 (161)  100 (127)  19 (270) | 975,434  975,434  874,602  135,237 | 1328 (136)  1328 (136)  1193 (136)  338 (250) |
| Moderna  Total, all doses  Dose 1 ^1^  Dose 2 ^2^  Dose 3 ^3^ | 160,365  160,365  144,642  19,606 | 23,094  12,010  10,780  304 | 26 (113)  12 (100)  13 (121)  NA | 133,637  133,637  119,492  16,037 | 148 (111)  148 (111)  116 (97)  18 (112) |
| AstraZeneca  Total, all doses  Dose 1 ^1^  Dose 2 ^2^  Dose 3 ^3^ | 91,560  91,560  85,996  0 | 13,359  6,901  6,457  NA | 28 (210)  16 (232)  12 (186)  NA | 75,741  75,741  70,994  NA | 153 (202)  153 (202)  131 (185)  NA |
| Johnson & Johnson  Total, all doses  Dose 1^1^ | 91,174  91,174 | 6,804  6,802 | 9 (132)  9 (132) | 81,786  81,786 | 37 (45)  37 (45) |
| 1 Including only dose 1, i.e. the total cohort  2 Including only dose 2, i.e. a subset of the cohort  3 Including only dose 3, i.e. a subset of the cohort  NA: Data are not shown due to cell counts bellow 5 | | | | | |

**Supplementary table 4** Incidence rates (95% CI) of VTE in the exposed and non-exposed period by sex, age category, vaccine type and vaccine brand.

|  | **n** | **Person-years exposed** | **Events in exposed period**  **(IR per 100,000 person-years)** | **Person-years non-exposed** | **Events in non-exposed period (IR per 100,000 person-years)** |
| --- | --- | --- | --- | --- | --- |
| ***All COVID-19 vaccines (including unknown), n = 2,133,853 (total cohort)*** | | | | | |
| All persons, 12-60 years  All persons, 60+ years  Male, all ages  Male, 12-60 years  Male, 60+ years  Female, all ages  Female, 12-60 years  Female, 60+ years | 1,429,641  704,212  1,053,702  715,798  337,904  1,080,151  713,843  366,308 | 201,002  121,554  156,642  98,681  57,961  165,914  102,321  63,583 | 215 (107)  331 (272)  280 (179)  107 (108)  173 (298)  266 (160)  108 (106)  158 (248) | 1,192,081  561,705  868,028  598,920  270,108  884,758  593,161  291,597 | 1056 (89)  1639 (292)  1356 (156)  504 (84)  852 (315)  1339 (151)  552 (93)  787 (270) |
| ***Vaccine type (mRNA versus vector), n = 1,951,894 persons with a homogeneous sequential vaccination regimen*** | | | | | |
| mRNA vaccines  All persons, 12-60 years  All persons, 60+ years  Male, all ages  Male, 12-60 years  Male, 60+ years  Female, all ages  Female, 12-60 years  Female, 60+ years | 1,217,573  551,568  870,958  612,693  258,265  898,183  604,880  293,303 | 175,981  96,999  133,012  87,798  45,214  139,968  88,183  51,785 | 177 (101)  276 (285)  226 (170)  88 (100)  138 (305)  227 (162)  89 (101)  138 (266) | 1,010,802  437,296  714,713  509,628  205,085  733,386  501,174  232,212 | 879 (87)  1365 (312)  1106 (155)  418 (82)  688 (335)  1138 (155)  461 (92)  677 (292) |
| Vector vaccines  All persons, 12-60 years  All persons, 60+ years  Male, all ages  Male, 12-60 years  Male, 60+ years  Female, all ages  Female, 12-60 years  Female, 60+ years | 127,603  55,152  100,208  70,462  29,746  82,547  57,141  25,406 | 12,204  7,961  10,618  6,321  4,297  9,548  5,883  3,664 | 22 (180)  15 (188)  23 (217)  13 (206)  10 (233)  14 (147)  9 (153)  5 (136) | 111,841  45,704  86,764  62,116  24,651  70,778  49,725  21,053 | 93 (83)  97 (212)  112 (129)  52 (84)  60 (243)  78 (110)  41 (82)  37 (176) |
| ***Vaccine brand (Pfizer/BioNTech, Moderna, AstraZeneca, Johnson & Johnson), n = 1,526,314 persons with a homogeneous sequential vaccination regimen*** | | | | | |
| Pfizer/BioNTech  All persons, 12-60 years  All persons, 60+ years  Male, all ages  Male, 12-60 years  Male, 60+ years  Female, all ages  Female, 12-60 years  Female, 60+ years | 955,723  227,492  582,937  478,000  104,937  600,278  477,723  122,555 | 135,537  36,640  84,109  67,228  16,881  88,168  68,309  19,859 | 132 (97)  126 (343)  118 (140)  61 (91)  57 (338)  140 (159)  71 (104)  69 (347) | 795,163  180,271  481,740  398,430  83,309  493,694  396,732  96,962 | 633 (80)  695 (386)  633 (131)  282 (71)  351 (421)  695 (141)  351 (88)  344 (355) |
| Moderna  All persons, 12-60 years  All persons, 60+ years  Male, all ages  Male, 12-60 years  Male, 60+ years  Female, all ages  Female, 12-60 years  Female, 60+ years | 153,209  7,156  85,210  81,705  3,505  75,155  71,504  3,651 | 22,139  955  12,295  11,826  467  10,799  10,313  485 | 19 (86)  7 (733)  14 (114)  10 (85)  NA  12 (111)  9 (87)  NA | 127,912  5,724  71,040  68,223  2,808  62,597  59,689  2,902 | 121 (95)  27 (472)  83 (117)  68 (100)  15 (534)  65 (104)  53 (89)  12 (414) |
| AstraZeneca  All persons, 12-60 years  All persons, 60+ years  Male, all ages  Male, 12-60 years  Male, 60+ years  Female, all ages  Female, 12-60 years  Female, 60+ years | 37,663  53,897  43,966  14,936  29,030  47,594  22,727  24,867 | 5,492  7,867  6,417  2,174  4,243  6,942  3,318  3,624 | 13 (237)  15 (191)  16 (249)  6 (276)  10 (236)  12 (173)  7 (211)  5 (138) | 31,171  44,570  36,349  12,344  24,005  39,393  18,827  20,565 | 56 (180)  97 (218)  88 (242)  28 (227)  60 (250)  65 (165)  28 (149)  37 (180) |
| Johnson & Johnson  All persons, 12-60 years  All persons, 60+ years  Male, all ages  Male, 12-60 years  Male, 60+ years  Female, all ages  Female, 12-60 years  Female, 60+ years | 89,924  1,250  56,227  55,515  712  34,947  34,409  538 | 6,710  94  4,199  4,146  53  2,605  2,564  40 | 9 (134)  NA  7 (167)  7 (169)  NA  NA  NA  NA | 80,657  1,130  50,406  49,763  643  31,374  30,887  487 | 37 (46)  NA  24 (48)  24 (48)  NA  13 (41)  13 (42)  NA |
| NA: Data are not shown due to cell counts bellow 5 | | | | | |

**Supplementary table 5** Incidence rates (95% CI) of VTE in the exposed and non-exposed period by risk group, vaccine type and vaccine brand.

|  | **n** | **Person-years exposed** | **Events in exposed period**  **(IR per 100,000 person-years)** | **Person-years non-exposed** | **Events in non-exposed period (IR per 100,000 person-years)** |
| --- | --- | --- | --- | --- | --- |
| ***All COVID-19 vaccines (including unknown), n = 2,133,853 (total cohort)*** | | | | | |
| Hormone-containing contraceptives or HRT yes  Hormone-containing contraceptives or HRT no  Malignancy yes  Malignancy no  Heart failure yes  Heart failure no  Chronic lung disease yes  Chronic lung disease no | 411,749  668,402  151,328  1,982,525  23,488  2,110,365  348,825  1,785,028 | 59,231  106,684  26,206  296,350  4,069  318,487  54,647  267,901 | 79 (133)  187 (175)  106 (404)  440 (148)  17 (418)  529 (166)  155 (284)  391 (146) | 341,137  543,621  120,528  1,633,257  17,984  1,735,801  284,735  1,469,051 | 348 (102)  991 (182)  520 (431)  2175 (133)  82 (456)  2613 (151)  716 (251)  1979 (135) |
| ***Vaccine type (mRNA versus vector), n = 1,951,894 persons with a homogeneous sequential vaccination regimen*** | | | | | |
| mRNA vaccines  Hormone-containing contraceptives or HRT yes  Hormone-containing contraceptives or HRT no  Malignancy yes  Malignancy no  Heart failure yes  Heart failure no  Chronic lung disease yes  Chronic lung disease no | 349,130  549,053  129,276  1,639,865  21,062  1,748,079  291,503  1,477,638 | 51,424  88,726  22,755  250,225  3,708  269,272  46,183  226,797 | 68 (133)  159 (179)  94 (413)  359 (143)  14 (378)  439 (163)  126 (273)  327 (144) | 288,201  445,185  102,529  1,345,570  16,064  1,432,035  237,365  1,210,733 | 306 (106)  832 (187)  456 (486)  1788 (133)  78 (486)  2166 (151)  619 (261)  1625 (134) |
| Vector vaccines  Hormone-containing contraceptives or HRT yes  Hormone-containing contraceptives or HRT no  Malignancy yes  Malignancy no  Heart failure yes  Heart failure no  Chronic lung disease yes  Chronic lung disease no | 34,516  48,031  8,086  174,669  1,112  181,643  25,591  157,164 | 3,486  6,061  1,063  19,101  150  20,015  3,197  16,969 | 8 (229)  6 (99)  NA  33 (173)  NA  36 (180)  5 (156)  32 (189) | 30,066  40,712  6,719  150,817  882  156,662  21,686  135,859 | 20 (67)  58 (142)  22 (327)  168 (111)  NA  189 (121)  37 (171)  153 (113) |
| ***Vaccine brand (Pfizer/BioNTech, Moderna, AstraZeneca, Johnson & Johnson), n = 1,526,314 persons with a homogeneous sequential vaccination regimen*** | | | | | |
| Pfizer/BioNTech  Hormone-containing contraceptives or HRT yes  Hormone-containing contraceptives or HRT no  Malignancy yes  Malignancy no  Heart failure yes  Heart failure no  Chronic lung disease yes  Chronic lung disease no | 271,426  328,852  61,105  1,122,110  10,534  1,172,681  185,837  997,378 | 38,792  49,376  10,005  162,271  1,688  170,589  27,629  144,648 | 54 (139)  86 (174)  51 (510)  207 (128)  6 (356)  252 (148)  66 (239)  192 (133) | 224,778  268,916  48,438  926,996  7,807  967,626  152,469  822,964 | 221 (98)  474 (176)  264 (545)  1064 (115)  50 (640)  1278 (132)  372 (244)  956 (116) |
| Moderna  Hormone-containing contraceptives or HRT yes  Hormone-containing contraceptives or HRT no  Malignancy yes  Malignancy no  Heart failure yes  Heart failure no  Chronic lung disease yes  Chronic lung disease no | 41,141  34,014  5,752  154,613  618  159,747  22,092  138,273 | 5,864  4,932  857  22,237  86  23008  3,188  19,906 | NA  9 (182)  6 (700)  20 (90)  NA  26 (113)  8 (251)  18 (90) | 34,312  28,269  4,689  128,948  468  133,168  18,341  115,296 | 29 (85)  36 (127)  19 (405)  129 (100)  NA  146 (110)  38 (207)  110 (95) |
| AstraZeneca  Hormone-containing contraceptives or HRT yes  Hormone-containing contraceptives or HRT no  Malignancy yes  Malignancy no  Heart failure yes  Heart failure no  Chronic lung disease yes  Chronic lung disease no | 12,763  34,831  6,549  85,011  1,061  90,499  18,196  73,364 | 1,870  5,072  946  12,411  147  13,212  2,641  10,716 | 7 (374)  5 (99)  NA  24 (193)  NA  27 (204)  NA  24 (224) | 10,573  28,819  5,317  70,414  835  74,905  15,007  60,721 | 15 (142)  50 (173)  22 (414)  131 (186)  NA  152 (203)  32 (213)  121 (199) |
| Johnson & Johnson  Hormone-containing contraceptives or HRT yes  Hormone-containing contraceptives or HRT no  Malignancy yes  Malignancy no  Heart failure yes  Heart failure no  Chronic lung disease yes  Chronic lung disease no | 21,751  13,196  1,537  89,637  51  91,123  7,392  83,782 | 1,616  989  116  6,687  4  6,800  553  6,250 | NA  NA  NA  9 (135)  NA  9 (132)  NA  8 (128) | 19,489  11,885  1,401  80,385  47  81,740  6,661  75,123 | 5 (26)  8 (67)  NA  37 (46)  NA  37 (45)  5 (75)  32 (43) |
| HRT: Hormone replacement therapy; NA: Data are not shown due to cell counts bellow 5 | | | | | |
